# Predicate Oriented Pattern Analysis for Biomedical Knowledge Discovery

**DOI:** 10.4236/iim.2016.83006

**Published:** 2016-05

**Authors:** Feichen Shen, Hongfang Liu, Sunghwan Sohn, David W. Larson, Yugyung Lee

**Affiliations:** 1 CSEE Department, University of Missouri, Kansas City, MO, USA; 2 Department of Health Sciences Research, Mayo Clinic College of Medicine, Rochester, MN, USA; 3 Department of Surgery, Mayo Clinic College of Medicine, Rochester, MN, USA

**Keywords:** Biomedical Knowledge Discovery, Pattern Analysis, Predicate, Query Generation

## Abstract

In the current biomedical data movement, numerous efforts have been made to convert and normalize a large number of traditional structured and unstructured data (e.g., EHRs, reports) to semi-structured data (e.g., RDF, OWL). With the increasing number of semi-structured data coming into the biomedical community, data integration and knowledge discovery from heterogeneous domains become important research problem. In the application level, detection of related concepts among medical ontologies is an important goal of life science research. It is more crucial to figure out how different concepts are related within a single ontology or across multiple ontologies by analysing predicates in different knowledge bases. However, the world today is one of information explosion, and it is extremely difficult for biomedical researchers to find existing or potential predicates to perform linking among cross domain concepts without any support from schema pattern analysis. Therefore, there is a need for a mechanism to do predicate oriented pattern analysis to partition heterogeneous ontologies into closer small topics and do query generation to discover cross domain knowledge from each topic. In this paper, we present such a model that predicates oriented pattern analysis based on their close relationship and generates a similarity matrix. Based on this similarity matrix, we apply an innovated unsupervised learning algorithm to partition large data sets into smaller and closer topics and generate meaningful queries to fully discover knowledge over a set of interlinked data sources. We have implemented a prototype system named BmQGen and evaluate the proposed model with colorectal surgical cohort from the Mayo Clinic.

## 1. Introduction

Researchers and health care practitioners prefer to conduct research in an evidence-based practice by using available research results when making decisions in health care. The main challenge we are facing to support evidence-based research is the big data problem along with large, complex, and dynamic medical data (e.g., clinical research data, EHRs, ontologies). A lot of medical ontologies and tools have been developed for biomedical research and applications. However, these are not sufficient for integrating or mapping unstructured data to structured data that will be significant for evidence-based research. It is mainly due to the lack of the ability to integrate data from such a variety of sources and extract both a cohesive structure and semantics from structured or unstructured data to support evidence-based research.

In order to extract a cohesive structure and semantics, it is essential to know what information exists and what significant relationships are among the related domains (e.g., discover genes responsible for a disease). Semantic Web is able to provide a platform of information exchange for biomedical knowledge. Increasingly, we are also seeing the emergence of biomedical and scientific collaborations. The first notable effort toward connecting scattered medical data is to materialize a data movement by the biomedical community (*i.e.*, Bio2RDF, OBO, LinkedCT) [1]. In addition, the Semantic Web Health Care and Life Sciences Interest Group (HCLSIG) [2] is aimed at utilizing Semantic Web technologies for innovative research and collaboration in the health care and life science domains. In this drive, large amounts of medical data have been specified and shared via machine-readable formats, such as the Resource Description Framework (RDF) and Ontology Web Language (OWL). The ontologies are developed to easily extend the work of others and share across different domains. These Semantic Web technologies make it easier and more practical to integrate, query, and analyze the full scale of relevant biomedical and healthcare data, as well as EHRs for cost effective health care systems [3].

However, to make seamless interoperability and interchange among heterogeneous datasets, there still exist significant difficulties. There are some existing promising semantic approaches for linking different datasets; however, they are computationally expensive and impractical for large scale ontologies since these works may still require human intervention. Furthermore, as the size of data increases drastically, it is difficult to discover information from structured/unstructured data in a single domain or cross domains, especially for those researchers with expertise in a specific domain. Thus, we need to reduce human intervention with the help of process automation in extraction and integration of semantics from structured or unstructured data.

For extraction of a cohesive structure and semantics from structured or unstructured data, identification of meaningful linking, either together within or across a large number of biomedical ontologies, is necessary. vSparQL is introduced to enable application ontologies to be derived from these large, fragmented sources such as the FMA [4]. A series of queries may be generated using large ontologies like the NCI thesaurus by extracting relevant information that is desired for applications [5]. The GLEEN project aims to develop a useful service for simplified, materialized views of complex ontologies [6]. However, these works are limited due to the lack of the comprehensive semantic analysis of large sources and the usage of the knowledge for query processing. We need to connect related information through a reference ontology that becomes a platform to link together multiple ontologies that cover a broad range of related information. Advanced techniques are needed to analyze these larger reference ontologies, rather than simply getting a slice of a reference ontology and applying it for a query process or decision support [5]. There is also some related work on using K-Means and Fuzzy C-Means for clustering microarray data [7] [8], but neither of them are concerned about the semantics of data nor hierarchical clustering.

In this paper, we applied RDF predicate oriented pattern analysis methodology and combined the advantages of Machine Learning with the added rigor of machine-readable semantics in extracting information and generating queries applicable for decision support in clinics [9]–[12]. The approach to converting free text to ontologies is based on text classification methods by identifying related information connected through relationships and classifying them according to their relevance. In addition, queries are evaluated by measuring the content of information, identifying possible extensions or compositions of queries and making a comparison with an existing query benchmark. We have implemented a prototype of the BmQGen system and evaluated the proposed query model based on the predicate oriented clustering with the colorectal surgical cohort from the Mayo Clinic.

## 2. Methods

The proposed framework BmQGen is based on the following steps: 1) extracting key words from report; 2) converting unstructured (free text) data to semantically structured (RDF/OWL) data; 3) arranging them into groups in a semantically meaningful manner; 4) generating queries for evidence-based practice; 5) providing visualization based query analytics tool.

**Figure 1:**
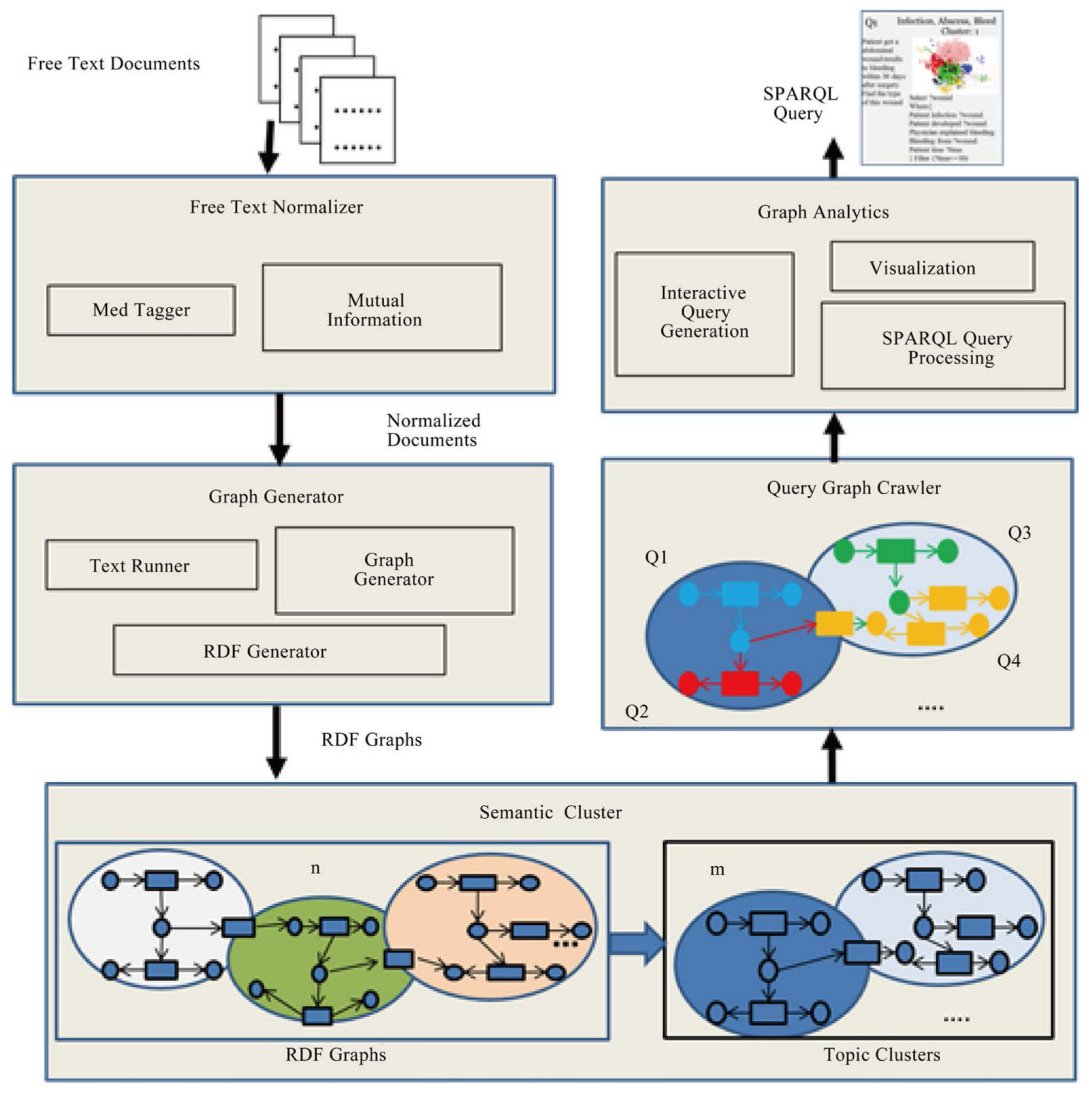
The BmQGen framework.

The main contribution of this paper is knowledge discovery from multiple domains by defining a predicate-oriented model for pattern-based fuzzy clustering, and processing cross-domain queries automatically generated from clustered patterns. Figure 1 summarizes the proposed system architecture. As seen in this figure, the Free Text Normalizer component first reads unstructured free text documents from doctors’ notes and patients’ records then uses MedTagger [13] to filter unnecessary terms out and convert free text terms to normalized ones. The Graph Generator component then applies TextRunner [14] on each unstructured normalized document and simplifies the term to generate a RDF triplet. The RDF/OWL data model [15] specifies resources (information on the entities and their relationship in the given document) in the form of triples <subject (S)-predicate (P)-object (O)>, where S denotes the resource, and P denotes aspects of the resource and expresses a relationship between S and O. In the Semantic Cluster component, many such triplets group *n* different ontologies, and these ontologies can be clustered into *m* semantic groups. The cluster graph is composed of the predicates either from a single domain or different domains. In the Query Graph Crawler component, semantically related queries will be generated for each of the *m* clusters crawling RDF/OWL (predicate) graphs by crawling the cluster graph to generate the query graph with one domain or across domains. The Graph Analytics component allows users to visualize the graphs and generate SPARQL queries through an interactive query interface for selected query graphs.

**Figure 2:**
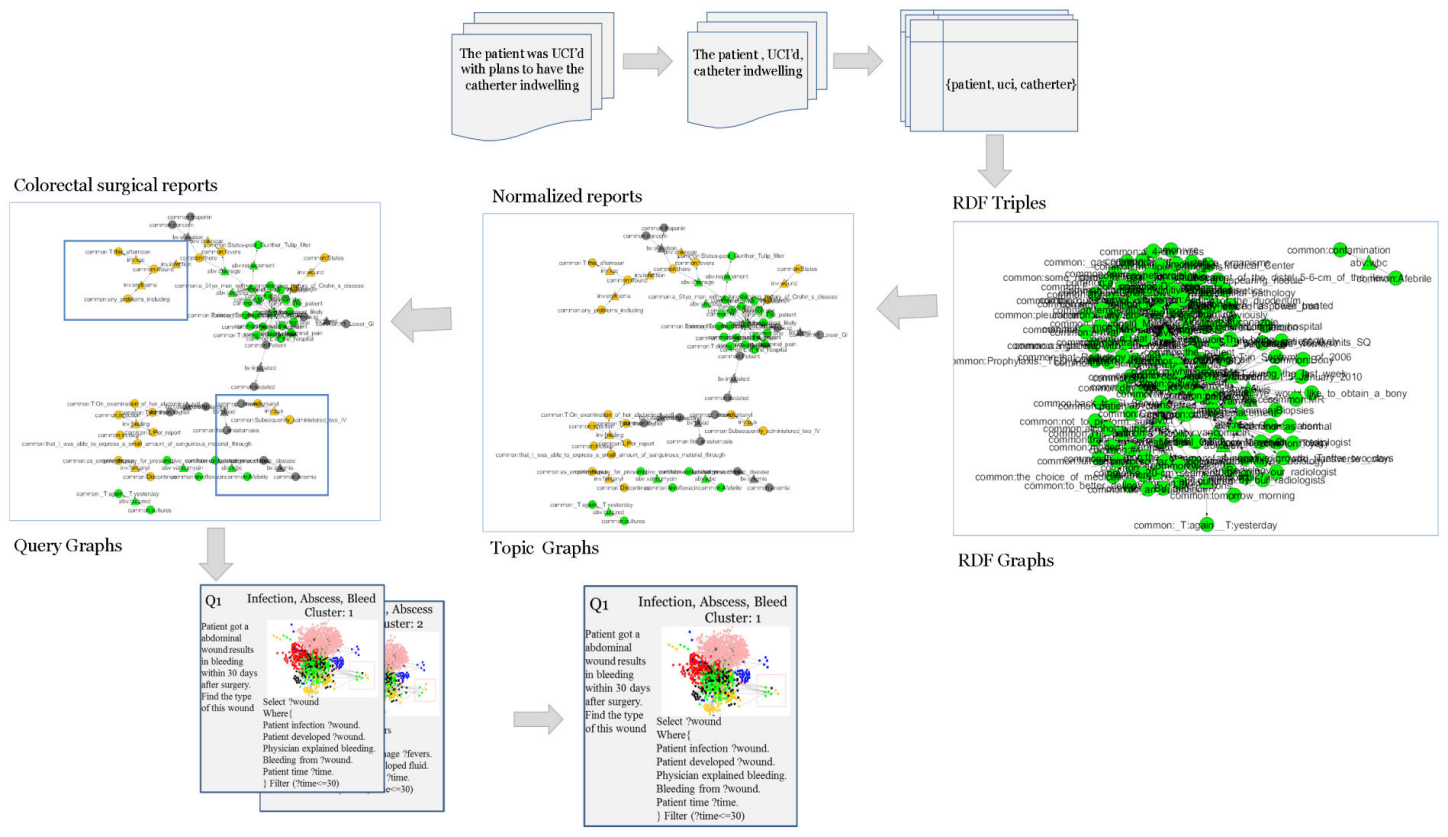
The BmQGen dataflow.

We first explain how to convert unstructured data to a linked data structure (RDF). We then present a fuzzy clustering algorithm to cluster the RDF/OWL graph. Finally, we evaluate the proposed model through the queries automatically generated from the clusters. The dataflow of the BmQGen framework is shown in Figure 2.

The basic steps of the BmQGen framework are described in the remainder of this section.

### 

#### Step 1: Feature Selection and Concept Annotation

We extracted various free text reports and then performed preprocessing to maintain terms consistently and exclude irrelevant terms, using filtering. Inequality of the likelihood holds between two different values to describe their correlations. To get the inequality of the likelihood between any pairs of words, we then extracted co-occurring terms from free text clinical notes, and calculated the inequality score to cluster them into a different category (domain). In this study, we used MedTagger, which is an open source concept detection and normalization tool through open health natural language processing. Specifically, this tool identifies phrases present in MedLex, a general semantic lexicon created for the clinical domain [16].

The point-wise mutual information was used to assess the inequality of the likelihood for given terms [17].

$$\text{Inequality}\left(c,o\right)=\log_{2}(N\left(c,o\right)*\log_{2}\frac{N\left(c,o\right)+0.01}{N\left(o\right)}-\log_{2}\frac{N\left(c\right)}{N}$$

*N* is the number of observations (e.g., the number of cases for all patients), *N*(*c*) is the number of cases having the concept *c*, *N*(*c*,*o*) is the concept c and the number of cases with a specific complication *o*, and *N*(*o*) is the number of cases with a specific complication *o*.

As the example showed in Figure 2, a given input text, “the patient was UCI’d with plans to have the catherter indwelling”, MedTagger recognized the uci and catheter concepts and we also found these terms come from ILEUS report. Then we used the point-wise mutual information measurement to calculate any inequality likelihood between these concepts (uci and catheter) and ILEUS. For example, the total number of cases is 1980, number of ILEUS cases is 400, the number of concept uci among all cases is 600, and the number of concept uci along with ILEUS cases is 500. Based on equation, the inequality between uci and ILEUS is 1.69. We did the same for all other concepts and ranked these concepts by their inequality score and only chose the top 60 of them. Then for any free text which contains top 60 concepts with inequality score, we used OpenIE [18] to get the triple <S-P-O> from the free text annotated with the concepts recognized by MedTagger. For this purpose, we first got a triple {the patient, UCI’d, catherter indwelling}. To make the triple normalized, we looked up the MedTagger concept in the dictionary again and converted the triple to {patient, uci, catherter}. For each complication case, we did the same work above in order to generate six ontologies, respectively.

In Table 1, we list some examples about how to map among clinical free text, MedTagger normalized terms and triplets.

#### Step 2: RDF Graph Construction

First, the top K concepts were selected and each sentence with these concepts in the datasets was annotated with the selected terms. We then extracted the assertions (RDF/OWL triples) from a given free test dataset considering the top K concepts of each domain and generated RDF/OWL triples, respectively. We used OpenIE to extract the triples from the free text. The OpenIE that is based on TextRunner, ReVerb [19] using (PoS) patterns, extracted a significant relation without any relation-specific input. OpenIE used a conditional random field (CRF) classifier to automatically extract triples representing binary relations (Arg1, Relation, Arg2) from sentences. The triples generated from OpenIE were connected to generate RDF graphs.

#### Step 3: Assertion Clustering and Query Generation

Our assumption for the predicate neighboring patterns (PNP) is that a predicate plays an important role in sharing information and connecting entities among heterogeneous data. For any given domain, the number of unique relations is much less than the number of concepts. Thus, this is another scalable approach for mapping domains than the concept-centric approach. A group of terms (subjects) can be connected to a group of terms (objects) through a single predicate unlike the concept-driven approach. From the unit of <subjects-predicate-objects>, a specific context can be discovered from the associated concepts (subjects, objects). From the neighbors of the predicates, a specific context can be discovered from the association of predicates and their subjects and objects. We can infer/predict missing predicates or missing concepts based on existing contexts. Therefore, we generated a hypothesis that when a graph can be clustered based on PNP patterns, data in the same clustered group have a closer relationship than when in different ones. Predicate neighboring patterns are important to link data together with a variety of domains.

## 3. Query Generation for Knowledge Discovery

### 3.1. Predicate Neighboring Patterns

A predicate P is representing a binary relation between two concepts (c1 and c2) in ontology. In RDF/OWL, P is represented as a property to express a kind of relationship (e.g., rdfs:subClassOf) between domain (subject) and range (object). The subject and object can be either from the same ontology or from different ontologies. In our study, relationships are defined by the empirical analysis of ontology data. We are particularly interested in predicates (relationships) that are different from existing approaches like PSPARQL [20] and SPARQLer [21]. Apart from being similar, predicates may share other aspects, e.g., sharing the same subjects or the same objects as well as the connectivity between predicates. This forces not only on concepts among graphs but also relationships of the concepts. In this paper, the two types of predicate patterns are defined as follows.

#### Share Patterns

As Table 2 shows, this pattern describes the resources sharing relationships between interacting concepts such as shared subjects or shared objects through the given relationship. Assume that two predicates are given as follows: P1 <Si, Oi> and P2 <Sj, Oj> where Si, Sj are a set of subjects and Oi, Oj are a set of objects in given ontologies.

#### Connection Patterns

According to Table 3, the connection pattern is a frequently recurring pattern with predicates observed during query processing as the basis for joining one query pattern to another. This pattern is based mainly on the connectivity of concept(s) through the respective predicates. This type of pattern describes the comprehension of the connectivity relationships between interacting predicates. Assume that two predicates are given as follows: P1 <Si, Oi> and P2 <Sj, Oj> where P1 is directly connected to P2 in the given ontologies Oi, Oj.

**Figure 3:**
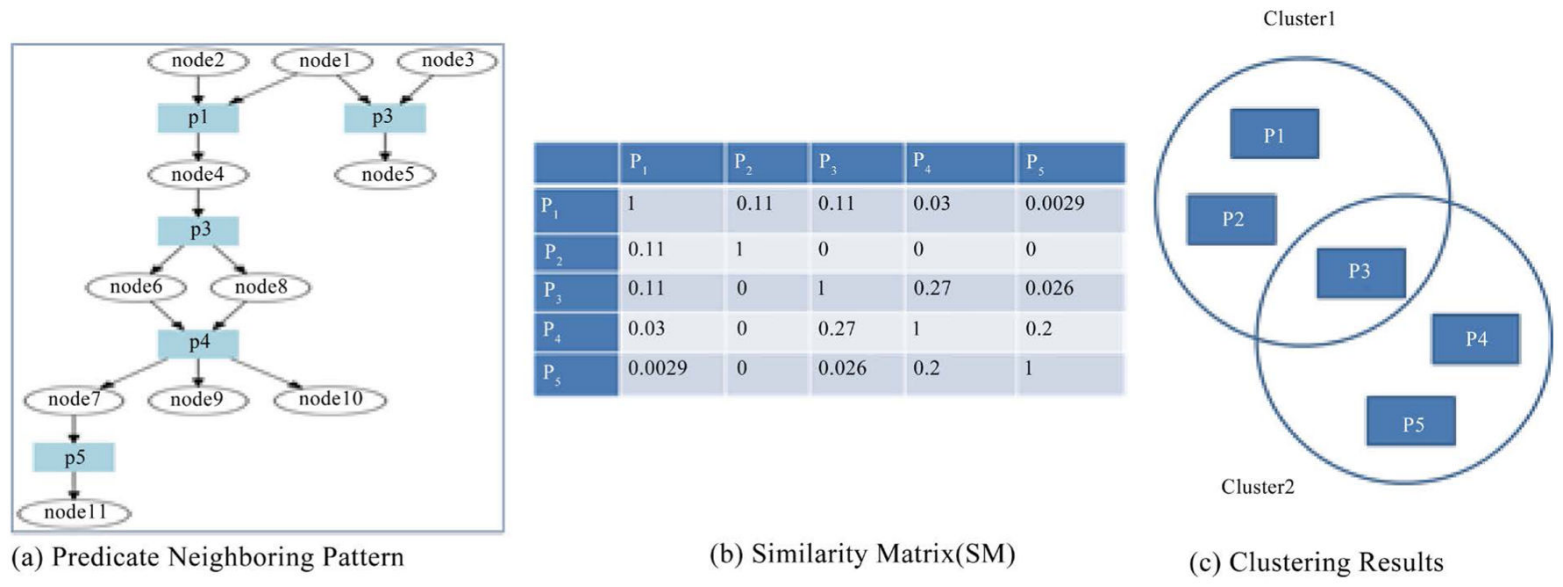
Predicate neighboring level and weighted similarity.

We gave the definition of predicate neighboring measurement in our previous paper [9]–[12]. Figure 3 gives an example to describe how neighboring pattern can be used to measure the closeness among predicates and how similarity matrix can be used to determine the clustering results. Figure 3(a) gives a RDF graph with 5 different predicates. In this graph, p1 and p2, p1 and p3, p3 and p4, p4 and p5 are in share patterns. Similarly, p1 and p4, p1 and p5, p2 and p3, p2 and p4, p2 and p5, p3 and p5 are in connection patterns. Based on different patterns each pair of predicates possesses, we calculated their similarity score and build the symmetric similarity matrix as Figure 3(b) shows. Then we applied Hierarchical Fuzzy C-Means (HFCM) clustering algorithm on this similarity matrix and built different clusters. As Figure 3(c) presents, p1, p2 and p3 are in cluster 1, p3, p4 and p5 are in cluster 2, specifically, p3 is the fuzzy predicate for cluster 1 and cluster 2.

### 3.2 Hierarchical Fuzzy C-Means Clustering

We posited that predicate clustering is a required step for efficient query processing involving the alignment and integration of ontologies. Here we clarify our approach to efficient query processing and query generation within the above theoretical framework. A query processing consists of a collection of several relationships between multiple properties. Given that properties are more closely related to some properties than others, property clustering and partitioning can be utilized for efficient query processing—the task of classifying a collection of properties into clusters. The guiding principle is to minimize inter-cluster similarity and maximize intra-cluster similarity, based on the notion of semantic distance.

To discover the neighboring relation between predicates, we used an innovative Hierarchical Fuzzy C-Means (HFCM) clustering algorithm. We extended a Fuzzy C-Means clustering algorithm [22] with a hierarchical approach applying a heuristic function. In general, we defined a fuzzy hierarchy for clustering setting a machine capacity threshold *α* to denote a certain number of triplets that each cluster for each level of the hierarchy can hold. The hierarchy can be constructed by applying the Fuzzy C-Means algorithm on each cluster until the number of triplets for each cluster is less than or equal to the threshold *α* or no further change of numbers of elements for each cluster can be made. To get the optimal number of clusters, we used Silhouette Width to evaluate results and chose the one with the biggest score. The HFCM algorithm is given in Algorithm 1.

**Algorithm 1:** Hierarchical fuzzy C-Means clustering (HFCM).

// *P* is an *n* × *n* predicate similarity matrix, *n* is the number of predicates in ontologies		
// *δ* is the threshold of silhouette width *C_ij_*		
Input: *P*, *δ*		
// a hierarchy with a set of clusters *C_ij_* the *j^th^* cluster at *i^th^* level		
Output: *C* = {*C*_11_,*C*_12_,···,*C_ij_*}		
1.	*i*=1	
2.	**repeat**	
3.	// *n* is the number of input predicates, *m* is the number of predicates of cluster *j* at level *i*,	
4.	// optimal *k* (*k*≤*m*≤*n*) from *m* predicates of the cluster at level *i* (*C_i_*) using *nsw*(*c_i_*) function	
5.	*sw*_1_ = *nsw*(*c_i_*) //compute Neighborhood silhouette width	
6.	*k* = Optimal K(*C_i_*,*sw*_1_)	//fine the optimal *k* based on *nsw*(*C_i_*)
7.	Change1 = false	
8.	**If** (k > 1) then	
9.	Change1 = true	
10.	**for** *j = 1 to k*	
11.	*μ_ij_* = *RM*(*p_j_*_1_, *p_j_*_2_,···, *p_jm_*) (// random mean for predicates in *C_ij_* (cluster *j* at level *i*)	
12.	**end**	
13.	**for each** *p_ij_* ∈ *P_i_* do	
14.	$\mu_{ij}=\mathrm{Argmin}\;w_{ij}^{m}\;\left(p_{ij},\mu_{ij}\right)j\in \{1,\cdots ,k\}//w_{ij}$ is the degree, m is the fuzzifier	
15.	**end**	
16.	Change2 = false	
17.	*sw* = 0	
18.	**repeat**	
19.	**for each** *μ_ij_* ∈ *U_i_* do	
20.	Update Cluster(*μ_ij_*)	
21.	**end**	
22.	**for each** *p_ij_* ∈ *P_i_* do	
23.	*NCen* = *Argmin*(*p_ij_*,*μ_ij_*) *j* ∈ {1,···,*k*}	
24.	**if** *NCen* ≠ *μ_ij_* then	
25.	*μ_ij_* = *NCen*	
26.	*C_ij_* = *C_ij_* ∪ *p_ij_*	
27.	Changed2 = true	
28.	**end if**	
29.	*sw*_2_ **=** Silhouette Width (*C_ij_*)	//computesil houette width
30.	**while** Changed2 == true	
31.	**while** Change1 == true and *sw*_2_ ≥ *δ*	

### 3.3. Query Generation

**Figure 4:**
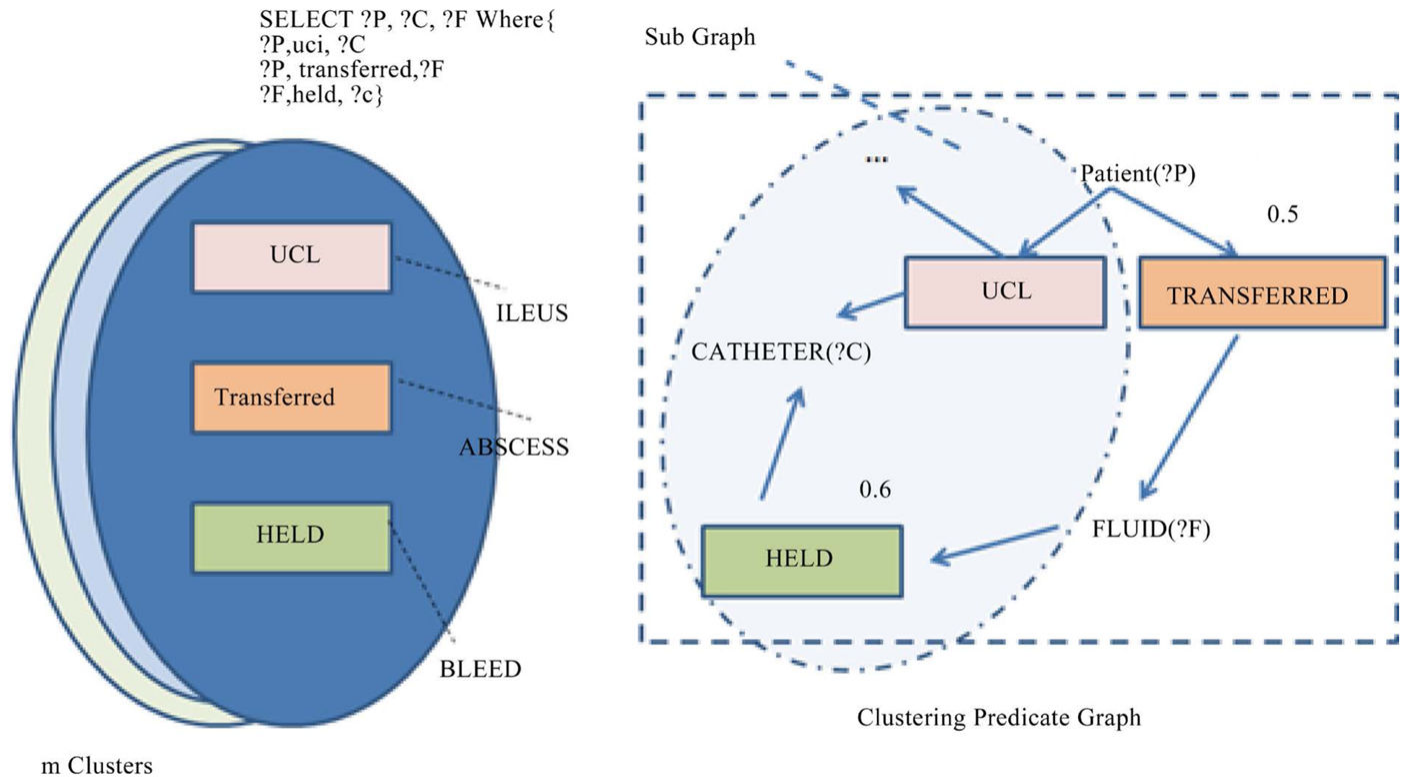
Clustering predicate graph for query generation.

From the HFCM clustering step, predicates are grouped into a number of clusters according to the similarity measurement of the predicate neighboring patterns (PNP). Each cluster will have a graph, called the cluster graph that is composed of the predicates either from a single domain or different domains. The Query Graph Crawler will take over and start crawling the cluster graph to generate the query graph with one domain or across domains. Figure 4 shows how the Query Graph Crawler generates queries: m clusters are generated after executing the HFCM algorithm and one of them has three predicates, namely uci from the ILEUS domain, transferred from the ABSCESS domain and held from the BLEED domain. As seen in Figure 4, we started to generate a query, first visiting the predicate that had the highest in-degree and out-degree and expanded it with its neighbors. In this example, we started with uci and then visited all its neighbors in a descending order of their similarity scores. As the similarity score between uci and held is 0.6, *uci visits held firs*t. And then uci visits transferred with the similarity score 0.5. After all uci’s neighbors were visited, we started to select its neighbor that had the highest similarity score. In this example, *held* takes turns to reach its neighbor and find *transferred*. The algorithm runs in an iterative way until there no more neighbors can be visited within the cluster. For example, if a query is generated with a predicate’s similarity score t ≥ 0.6, then the generated graph will include only uci and held. Finally, a SPARQL query will be generated by replacing the names of subjects and objects with variables.

## 4. Results and Discussion

### 4.1. Specification

The BmQGen prototype system was implemented using Java in an Eclipse Juno Integrated Development Environment [23]. Apache Jena API [24] was used to parse OWL/RDF datasets and retrieve triple information. We used the R computing environment [25] for our experimental validation. We implemented a software plugin for query and schema graph visualization using CytoScape 3.0.2 [26]. In addition, we set the machine capacity threshold *α* as 1/3 of the total size of the triples.

### 4.2. Case Study

As a case study, the Mayo Clinic’s colorectal surgical reports were used to generate queries by categorizing relationships among six colorectal postsurgical complications (deep vein thrombosis/pulmonary embolism, bleeding, wound infections, myocardial infraction, ileus and abscess/leak). Postsurgical complications are related to general or certain type of surgeries. Clinical data of six complications after colorectal surgery were attempted, analyzed to find interesting patterns/associations in a single or multiple complications, and generated comprehensive cross-domain queries that might be useful in conducting evidence-based practice by using available research results. We assume six postsurgical complications represent six domains. Predicate profiles and association patterns are important to link data together with a variety of domains.

### 4.3. Convert Colorectal Surgical Cohort to Ontology

Our case study has 1980 colorectal surgical cases for 1416 patients between 2005 and 2013 enrolled at the Mayo Clinic in Rochester, MN. We used our previous work, MedTagger to extract concepts from clinical notes written about any complications within 30 days after surgery in cohort. The top 60 concepts (by their inequality scores) were used for information extraction. The definition of the 6 complications is shown in Table 4.

**Figure 5:**
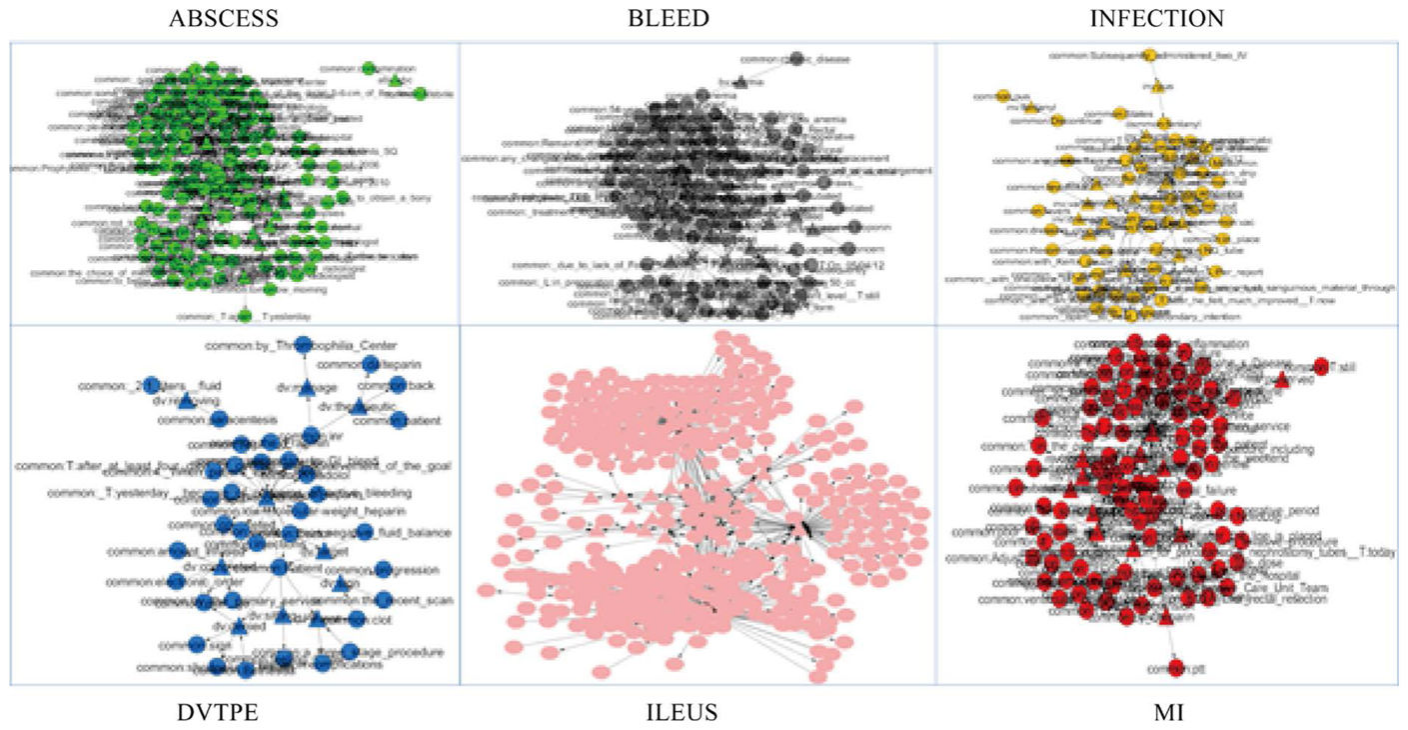
Visualization of 6 complication ontologies.

We built six ontologies based on the top 60 terms ranked by their inequality scores. Figure 5 gives visualizetions for each of the six ontologies. We used different colors to indicate different domains, so that ABSCESS is in green, BLEED is in gray, DVTPE is in blue, ILEUS is in pink, MI is in red and INFECTION is in orange. Table 5 shows the statistics of each ontology. Among the six ontologies, there are a total of 445 subjects, 83 predicates, 482 objects and 1210 triples involved. We then integrated six ontologies together to make them interlinked and prepared to apply a Hierarchical Fuzzy C-Means clustering algorithm on it.

### 4.4 Hierarchical Fuzzy C-Means (HFCM) Clustering Approach

**Figure 6:**
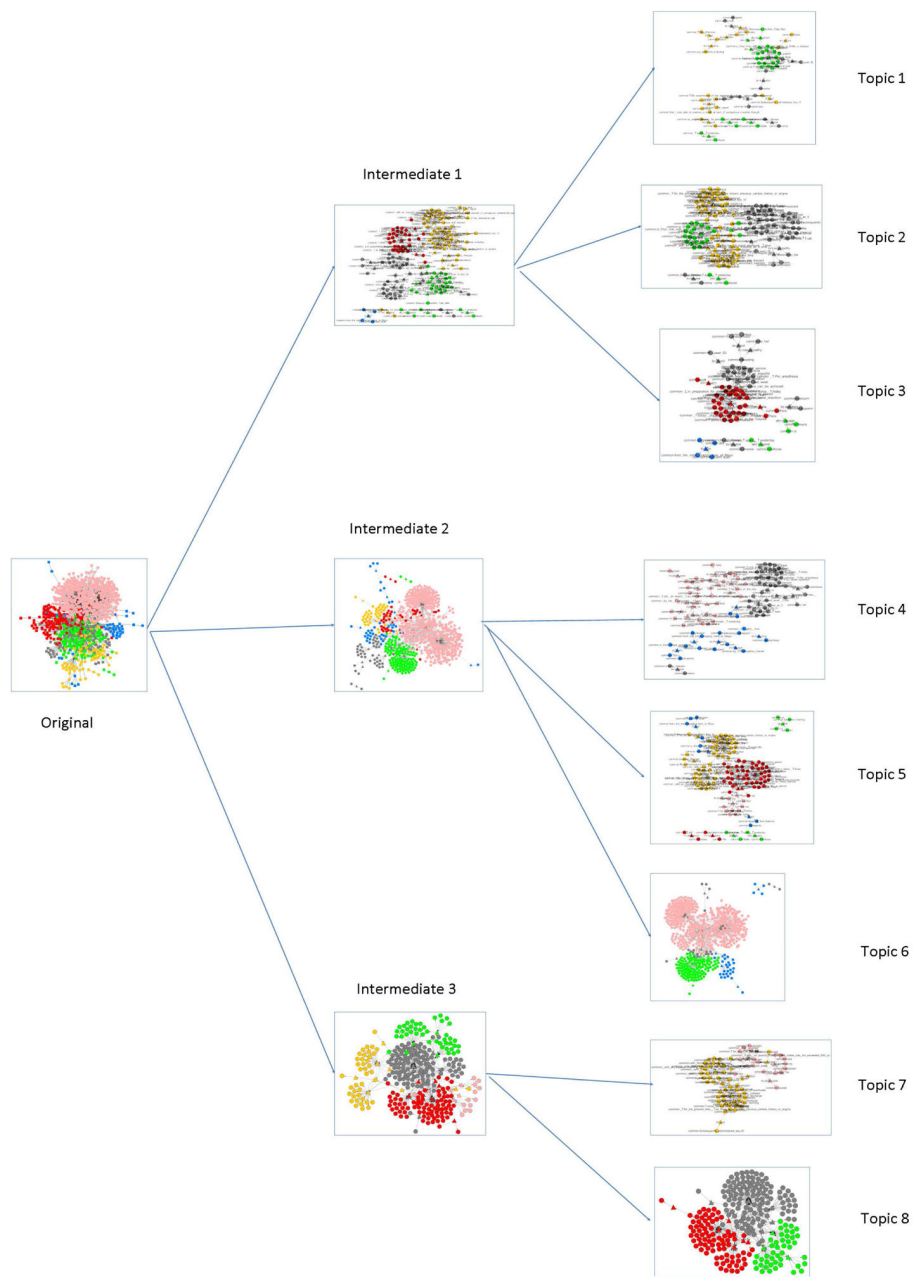
Visualization of hierarchical fuzzy C-Means clustering.

We applied Hierarchical Fuzzy C-Means (HFCM) clustering for integrated ontology on an input predicate similarity matrix with size 83 × 83. As a result, we got eight different topics. The hierarchical clustering graph is showed in Figure 6. The original integrated ontology was partitioned into three intermediate sub-topics based on the optimal Silhouette Width. In addition, three intermediate sub-topics can be further split into eight smaller topics with the best Silhouette Width. Because eight topics cannot be further clustered, then BmQGen stopped the HFCM algorithm and produced eight topics as the final output. We collected the top five predicates for each topic by their total in-degrees and out-degrees and summarized each topic with those predicates. What is more, out of the top five ranked predicates, we also selected top two unique predicates with the most in-degrees and out-degrees for each topic. Unique predicates indicate those predicates that appear in only one topic. Therefore, some topics have unique predicates but some do not. Based on the top predicates and unique predicates, we generated a signature for each topic to summarize the content of each topic.

**Figure 7:**
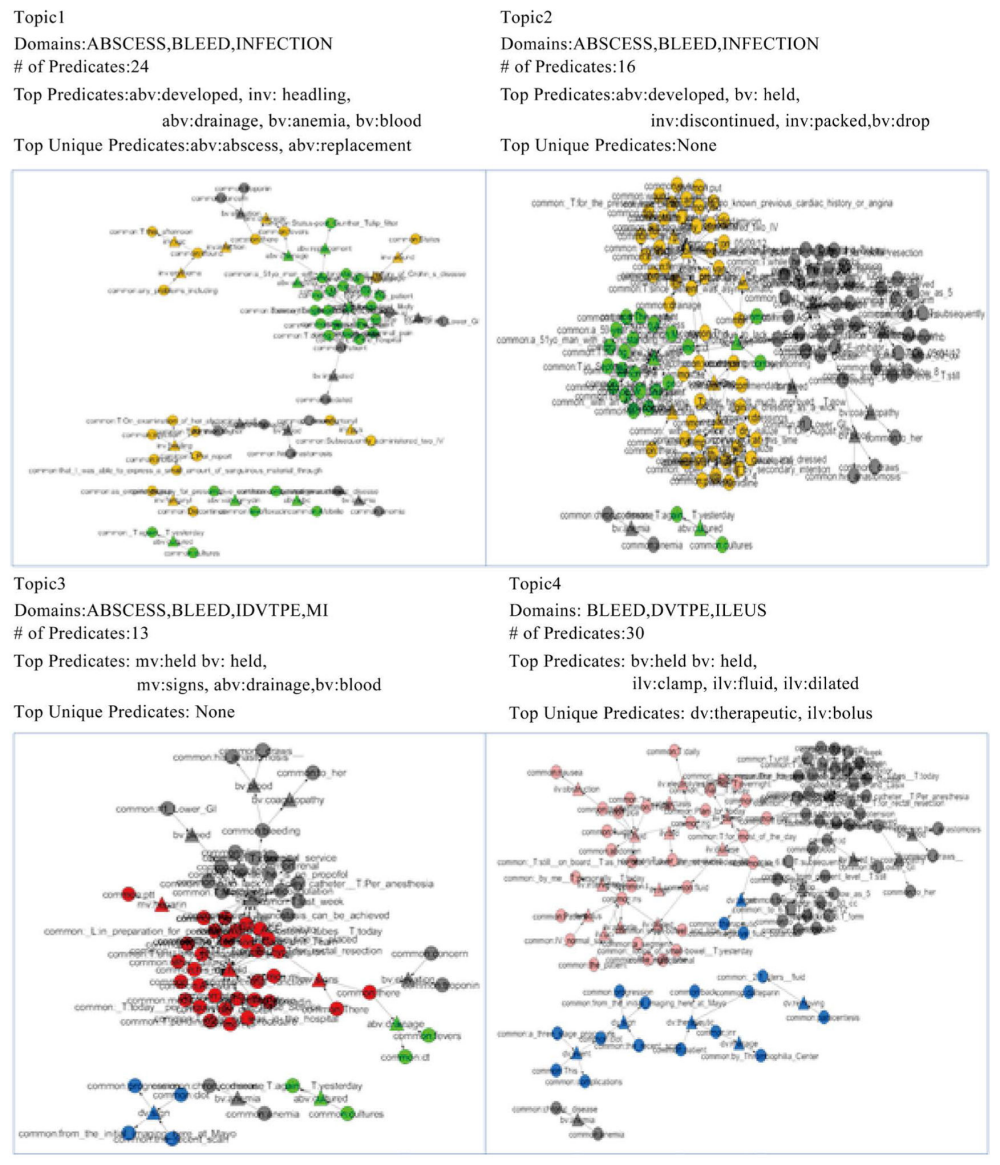
Detailed information for Topics 1–4.

Figure 7 shows topics 1–4. Topic 1 includes 3 complications (ABSCESS, BLEED and INFECTION) with 24 predicates in total. The top five predicates for Topic 1 are *abv: developed*, *inv: healing*, *abv: drainage*, *bv: anemia* and *bv: blood*, and the top two unique predicates for Topic 1 are *abv: abscess* and *abv: replacement*. By analyzing the signature of Topic 1, we found that Topic 1 describes the close relationship among drainage, anemia, blood and incision healing. Similar to Topic 1, Topic 2 also covers three complications (ABSCESS, BLEED and INFECTION) with 16 predicates. Top 5 predicates for Topic 2 are *abv: developed*, *bv: held*, *inv: discontinued*, *inv: packed* and *bv: drop*, and there are no unique predicates for Topic 2. From this signature, Topic 2 explains that the drop of some life indicators (e.g., hemoglobin) for a patient may be related to the complication (e.g., abscess) developed by such patient; the patient’s wound infection is discontinued for the reason that the infection area is packed with gauze. Topic 3 introduces four complications (ABSCESS, BLEED, DVTPE and MI) with 13 predicates. Top 5 predicates for Topic 3 are *mv: held*, *bv: held*, *mv: signs*, *abv: drainage* and *bv: blood*, and the unique predicates for Topic 3 are none. This signature illustrates BLEED and MI might hold the same symptoms, which are also related to drainage and blood. Topic 4 mentions three different complications (BLEED, DVTPE and ILEUS) with 30 predicates. The top five predicates for Topic 4 are *bv: held*, *bv: drop*, *ilv: clamp*, *ilv: fluid* and *ilv: dilated*, and the top two unique predicates for Topic 4 are *dv: therapeutic* and *ilv: bolus*. From this signature, we conclude that fluid has a potential relationship with the dilated, drop of life indicator and ng tube; therapeutic is associated with bolus.

**Figure 8:**
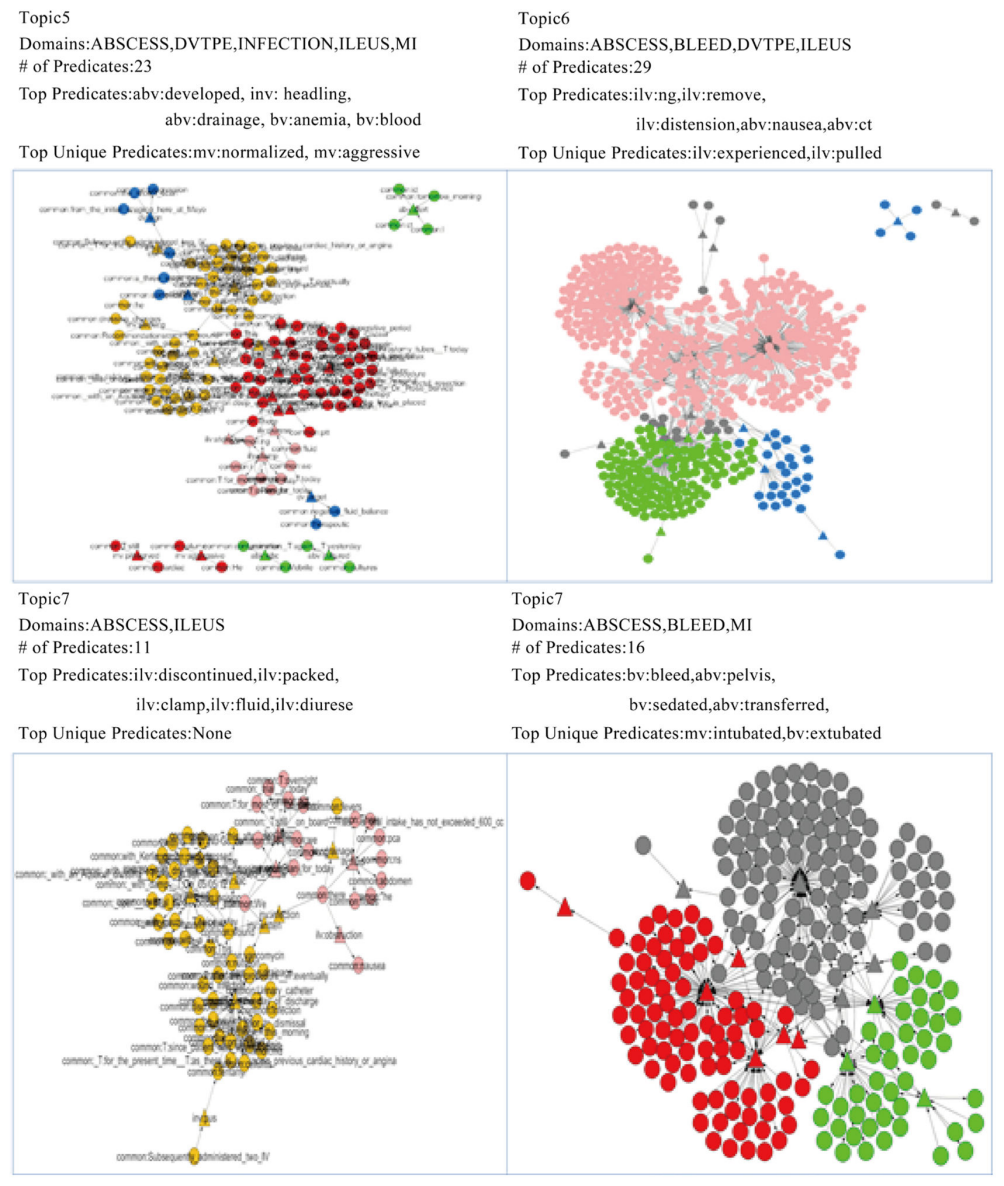
Detailed information for Topics 5–8.

Figure 8 shows topics 5–8. Topic 5 describes 5 complications (ABSCESS, DVTPE, INFECTION, ILEUS and MI) with 23 predicates. The top 5 predicates for Topic 5 are *abv: developed*, *inv: healing*, *abv: drainage*, *bv: anemia* and *bv: blood*. The top 2 unique predicates for topic 5 are *mv: normalized* and *mv: aggressive*. The top 5 predicates for Topic 5 convey the exact same information as Topic 1 does. However, unique predicates from Topic 5 tell us that the patient’s pain remained poorly-controlled even with an aggressive multimodal; meanwhile, the patient’s hypotension had normalized systolic pressure. Topic 6 indicates 4 complications (ABSCESS, BLEED, DVTPE and ILEUS) with 29 predicates. The top 5 predicates for this topic are *ilv: ng*, *ilv: remove*, *ilv: distension*, *abv: nausea* and *abv: ct*. The top 2 unique predicates are *ilv: experienced* and *ilv: pulled*. This signature summarizes the scenario that a patient’s ng tube was pulled out, and this patient also felt nausea and distension. Topic 7 covers 2 complications (ABSCESS and ILEUS) with 11 predicates. The top 5 predicates under Topic 7 are *inv: discontinued*, *inv: packed*, *ilv: clamp*, *ilv: fluid* and *ilv: diurese*. The top 2 unique predicates are none. This topic is also very similar to Topic 2 and Topic 4 but with more information on diuresis. Topic 8 is related to 3 complications (ABSCESS, BLEED and MI) with 16 predicates. The top 5 predicates involved in this topic are *bv: bleed*, *abv: pelvis*, *bv: sedated*, *abv: transferred* and *abv: read*. The top 2 predicates are *mv: intubated* and *bv: extubated*. This topic describes the bleeding situation of the patient’s pelvis; such patient was sedated; intubated and extubated operations were also applied on this patient.

**Figure 9:**
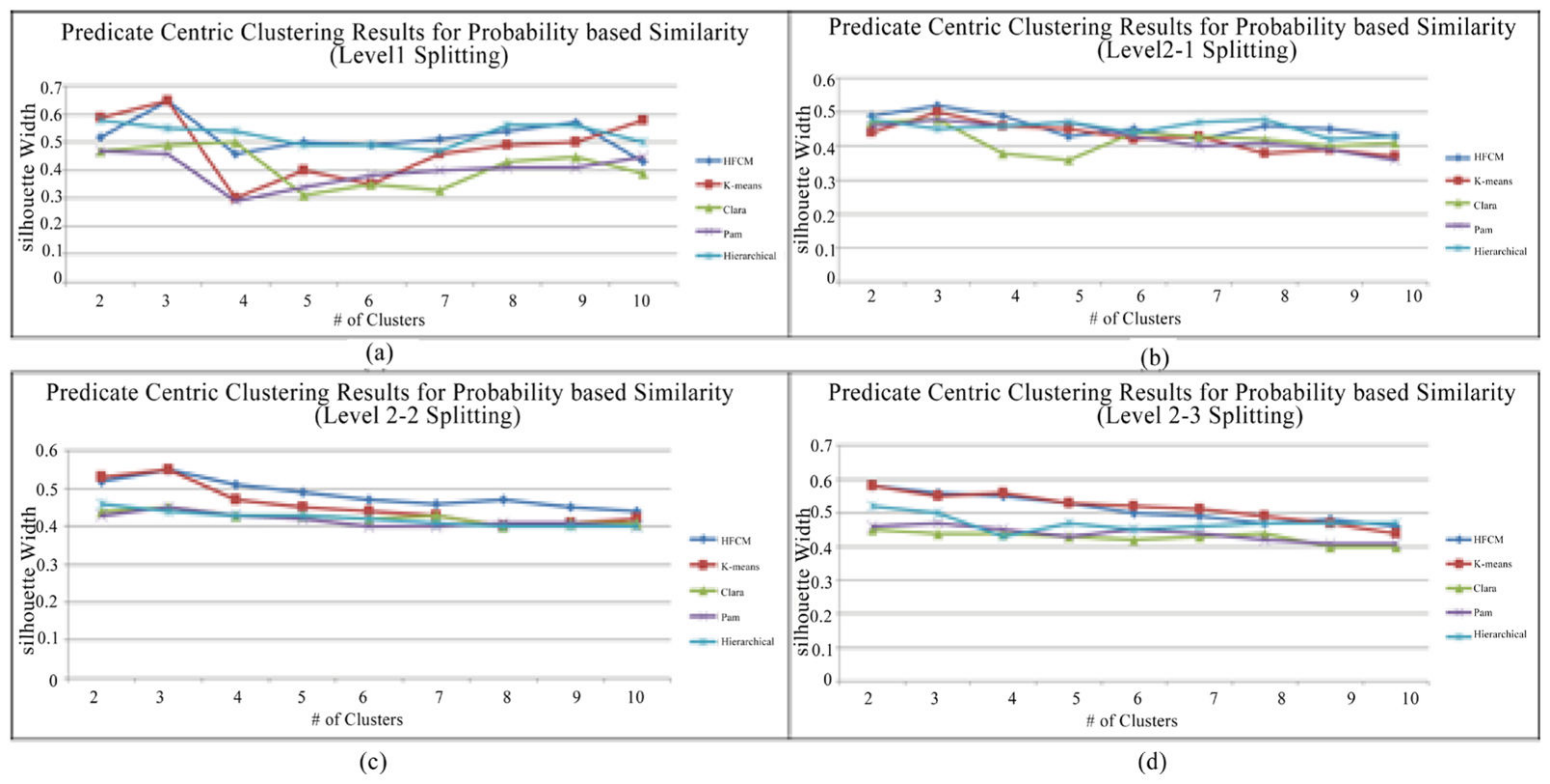
Predicate oriented clustering decision making on different levels.

We also conducted an experiment among different clustering algorithms to validate that HFCM is the optimal approach to do topic discovery. Silhouette width is a method of validation of consistency within clusters of data. Figure 9 shows validation for four partitions for each level (one first level and three second levels) with five different clustering algorithms (Hierarchical Fuzzy C-means [22], K-means [27], Clara [28], Pam [29] and Hierarchical Clustering [30]) on the similarity matrix. Figure 9(a) shows the splitting from the original ontology to intermediate clusters. Clara, Pam and Hierarchical clustering algorithms showing a relatively stable Silhouette Width for many cases and could not find an optimal cluster number. Both HFCM and K-means give the highest Silhouette Width 0.65 when the cluster number is 3. That shows the reason why the original ontology is split into three intermediate clusters. Similarly, Figures 9(b)–(d) present highest Silhouette Width for level 2-1, level 2-2 and level 2-3 splitting, which are 0.52, 0.55 and 0.58 with HFCM, respectively. This explains the reason why the Intermediate 1 Cluster is split into 3 clusters, Intermediate 2 Cluster is split into three clusters and Intermediate 3 Cluster is split into two clusters.

### 4.5. Validation of Clustering Results with Golden Standard

**Figure 10:**
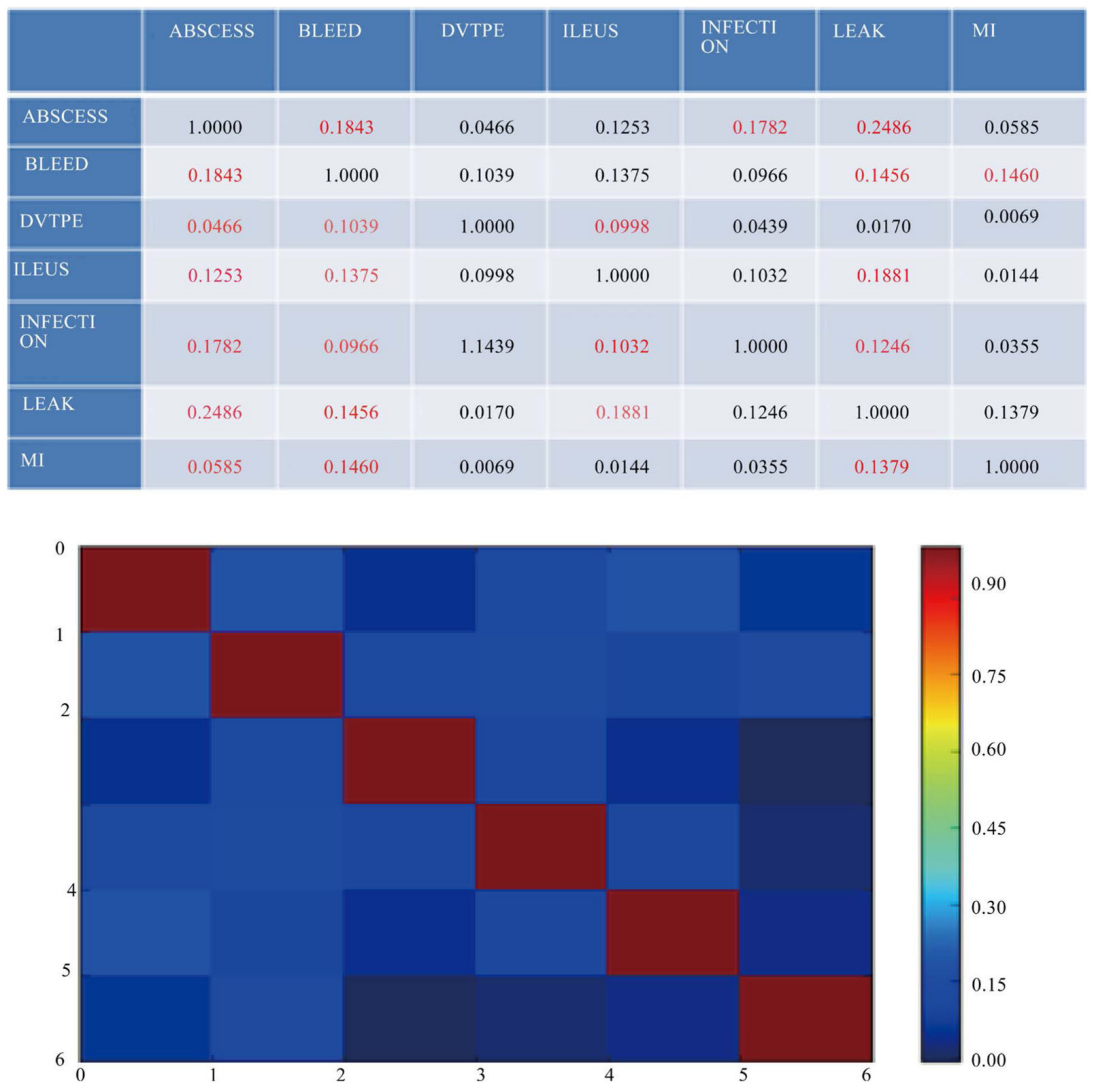
Correlation matrices for golden standard.

For those eight generated topics, we used a golden standard file provided by a medical expert, Dr. David W. Larson, in the Colon and Rectal Surgery department at the Mayo Clinics to validate our clustering output. This file lists indications of seven types of complications for 1505 patients after colorectal surgery from 2005 to 2013. In our study, we considered six types of complications by treating ABSCESS and LEAK as the same complication (unlike the golden standard) for the sake of simplicity. A patient may have had no complications or up to seven complications as the golden standard specified. We built correlation metrics based on this benchmark to find out which complications showed a strong positive correlation. Figure 10 represents the matrices with visualization. The number in red represents the top 3 relative strongest correlations for each complication. It is obvious to see that the complications ABCESS, BLEED and INFECTION have a relative stronger correlation than other complications that verifies that Topic 1 and Topic 2 are valid. ILEUS has a relative stronger relationship with ABSCESS and BLEED, verifying that Topic 6 is valid. LEAK and ILEUS are also strongly associated, verifying that Topic 7 is valid. MI is strongly related to ABSCESS and BLEED, and we can also verify that Topic 8 is valid. DVTPE does not have a very strong relationship with other complications, but this weak correlation with ILEUS, BLEED and ABSCESS is captured by Topics 3, 4, 5 and 6. Therefore, the clusters we generated by the HFCM follow the same correlation provided by the golden standard benchmark.

### 4.6. Query Generation and Visualization

**Figure 11:**
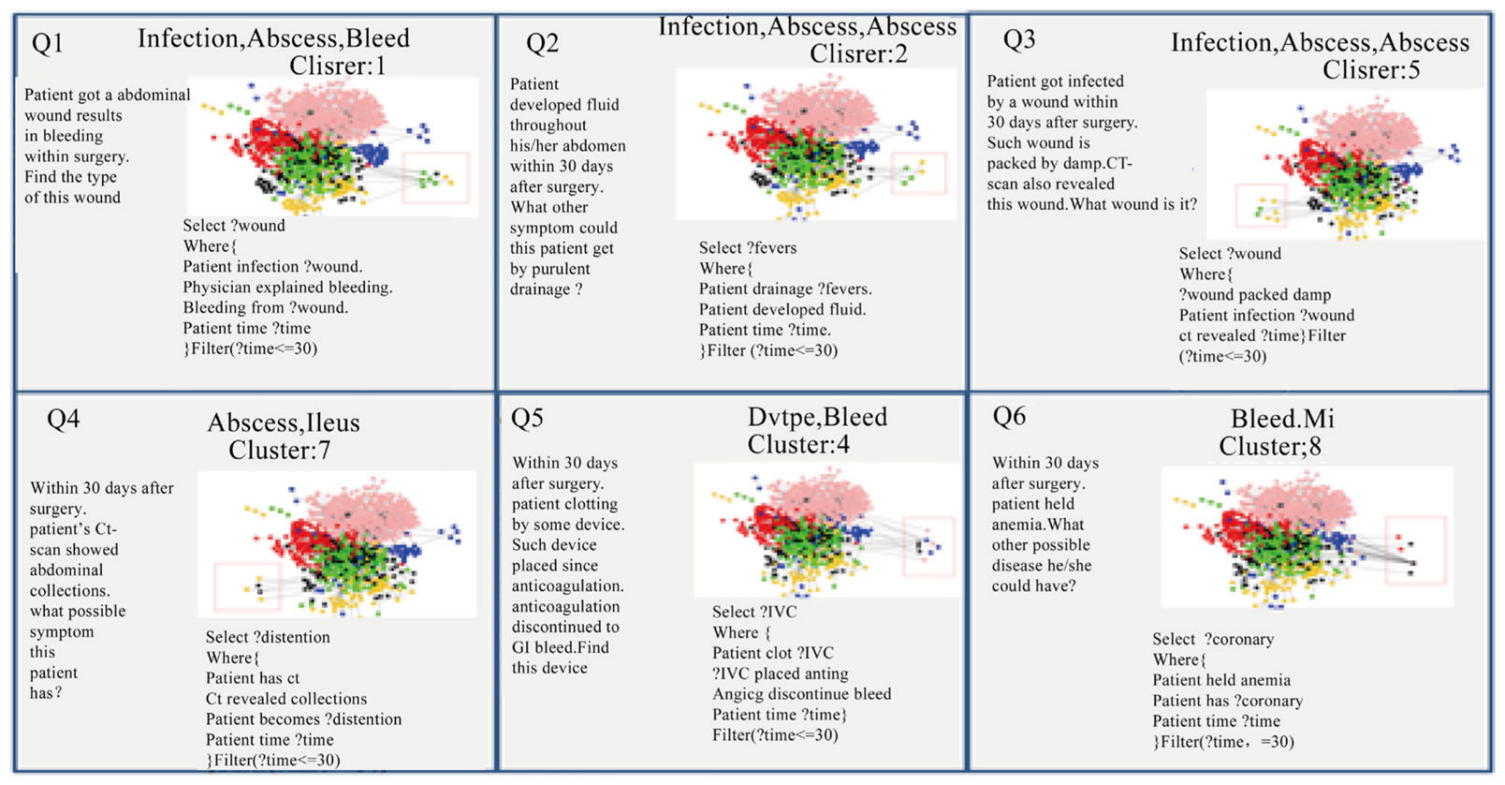
Cross complications queries.

**Figure 12:**
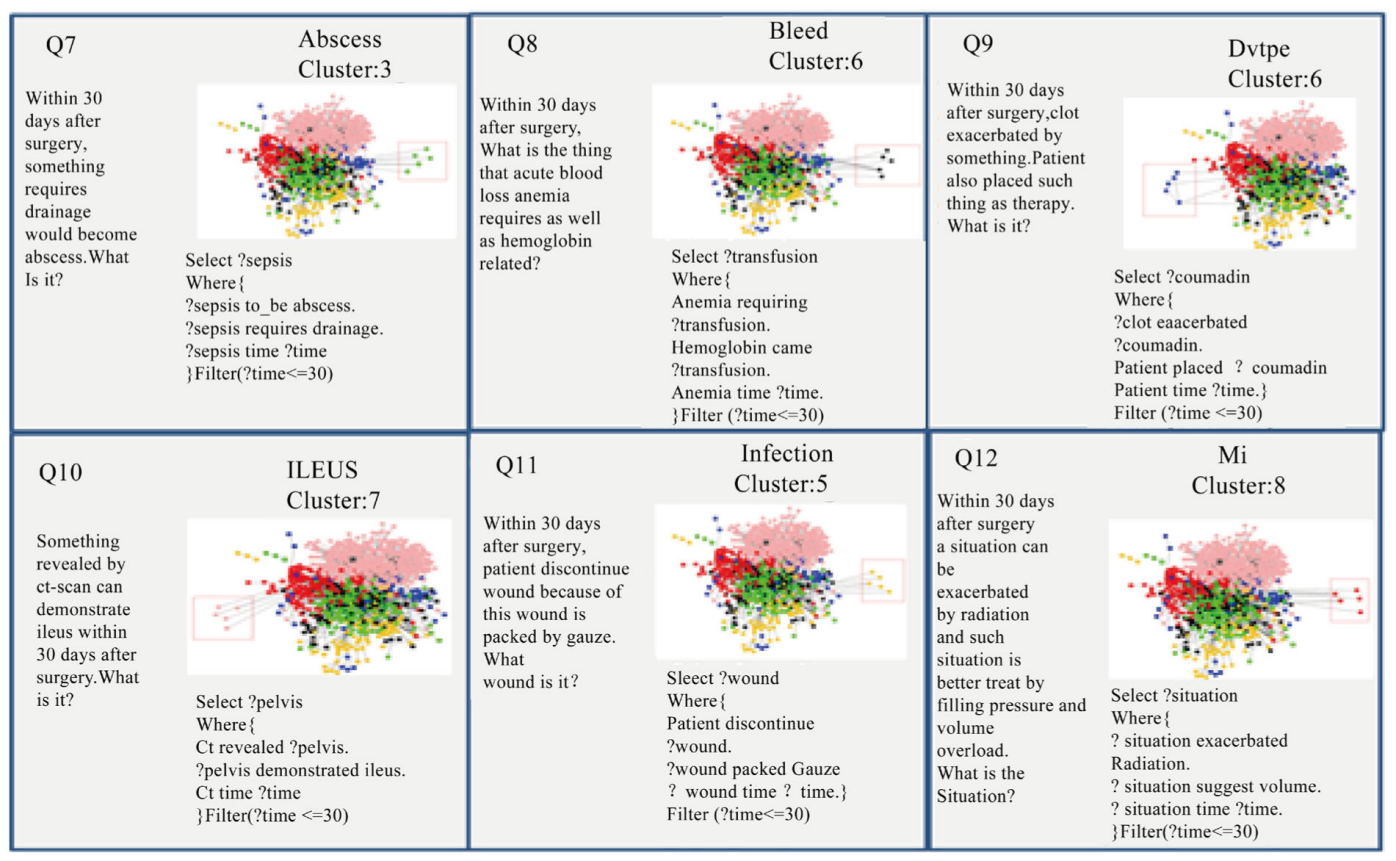
Single complication queries.

The SPARQL queries we generated for each cluster are sown in Figure 11 and Figure 12. For the predicate graphs across six domains, we used a rectangle to identify the query boundary out of the whole predicate graph. Queries 1 to 6 are cross domain queries that are automatically generated from each cluster. These queries identify the relationships among different post-surgical complications. For example, INFECTION, ABSCESS and BLEED are closely related to each other through the predicates of *wound*, *bleeding* or *fever*. DVTPE and BLEED are usually related through the predicate *clot*. ABSCESS and ILEUS are usually related to each other through *abdominal collections* and *distention*. MI and BLEED are closely related to each other through *anemia* and *coronary*. Queries 7 to 12 are about queries within a single complication. We also find some interesting query patterns for each of the six complications. For instance, in ABSCESS, *sepsis* usually comes with *drainage*. In BLEED, *transfusion* connects to *anemia* and *hemoglobin*. In DVTPE, *coumadin* and *clot* occur together. In ILEUS, *ct scan* and *pelvis* have a close relationship. In INFECTION, *patients* discontinue *wound* after the wound be packed. In MI, pressure and volume overload can be a good treatment for a problem exacerbated by radiation.

### 4.7. Discussion

Many efforts exist on semantic annotation of data and knowledge discovery on biomedical data [31]–[34]. Especially for [31], the authors annotated a different knowledge domain into their ontology. Moreover, [31] provided a solution to extract existing links among nodes and is capable of predicting potential links or missing links between source and destination. Compared to these works, our research puts more effort on the predicates’ relationship detection instead of on the concept nodes.

Furthermore, various data normalization and integration frameworks have been built to complete single domain, as well as cross domain, knowledge discovery. Commonly used medical ontologies are Bio2RDF [35], TMO (Translational Medicine Ontology) [36], Chem2Bio2RDF [37], SIO (Semanticscience Integrated Ontology) [38], ATC (Anatomical Therapeutic Chemical) and DrugBank integration [39], Chem2Bio2OWL [40], Linked Life Data [41], Linked open drug data LODD [42] and LinkedCT: A Linked Data Space for Clinical Trials [43]. These datasets provide a convenient and efficient way for researchers to explore and retrieve valuable information. Similar to these works, we also normalized terms and built our own ontology. However, we used an inequality of likelihood to select top relevant terms and then built ontologies based on these terms.

Meanwhile, a variety of research has been conducted to do systematical and computational knowledge discovery for cross domain knowledge. Specifically, HeteSim [44] and [45] are general systems for relationship extraction and linking detection for heterogeneous datasets. iPHACE [46] investigated a model to acquire knowledge between drug-target interactions. ChemProt [47] provided a database to discover relationships between disease and chemical biology. STITCH 3 [48] retrieved information between chemicals and proteins. In [49], the authors built an integrated platform of drugs, targets and clinical outcomes aimed at supporting drug repurposing. Kinnings *et al*. [50] were able to discover a relationship between drug and disease by deploying chemical systems biology. Campillos *et al*. [51] were able to identify a drug target by using side-effect similarity, and thus found association among the drug, target, and side effect. The Connectivity Map [52] made the use of gene-expression signatures to discover a relationship among small molecules, disease, genes and drugs. In our work, we applied an innovative, unsupervised learning algorithm on a similarity matrix and partitioned the original datasets into several meaningful topics.

Some research focuses on predicate based mining. Shi and Weninger [53] provided a predicate oriented path finding approach to do fact checking in a large knowledge graph. VEPath Cluster [54] proposed a combination of a vertex-centric and edge-centric approach for metapath graph analysis to enhance the clustering quality of cross domain datasets. In addition, pattern analysis was also widely used in data mining and knowledge discovery. In paper [55], the author proposed an interactive way to do data mining by applying pattern mining. In paper [56], the authors used electronic health record data as a use case to introduce an approach to perform data mining and visual analysis on clinical event patterns. WHIDE [57] is a tool for colocation pattern mining in multivariate bio-images. Huang *et al*. [58] accomplished the goal of clinical pathway pattern discovery by using probabilistic topic models. Lasko *et al*. [59] introduced a computational phenotype pattern discovery with unsupervised learning on clinical data. Our research focuses on predicates oriented pattern analysis. In BmQGen, we used our defined predicate neighborhood pattern measurement to quantify the closeness relationship among predicates in RDF.

The limitation in this work is that the datasets we used in this paper were quite noisy and some false positive and true negative cases exist in the mapping between words/phrases and complications. Therefore, the inequality metrics generated by the previous work may not represent the true inequality of the likelihood. What is more, although we built six ontologies, they are mixed with both instances and schemas, so contents within ontologies are not in an abstract level, therefore, clustering results and query outputs will be refined if a more accurately normalized input is given. To provide a better solution, words/phrases identified with a high inequality of likelihood need to be selected to get more normalized data. We also need to try different ontologies and dictionary tools to acquire a better annotated dataset for query generation.

## 5. Conclusions

In this study, we presented the idea of predicate based pattern analysis, investigated the use of ontology and applied an unsupervised machine learning approach to integrate a heterogeneous unstructured resource with a semi-structured knowledge base. In application level, we achieved specific topic based pattern analysis as well as query generation for cross domain knowledge discovery. A BmQGen framework was proposed to process any RDF/OWL datasets from heterogeneous resources. For the evaluation purpose, we adopted a case study with colorectal postsurgical complications and demonstrated that the BmQGen framework was capable of extracting a cohesive structure and semantics, as well as interesting patterns from structured/unstructured complication datasets. By using the colorectal surgical reports from the Mayo Clinic and golden standard, we successfully validated our clustering results, thereby providing solid evidence for automatic query generation.

In future work, we will improve the accuracy of information extraction by refining the data normalization workflow with different ontologies and extraction/normalization tools (e.g., MetaMap [60]). We will also get more support from domain experts to improve the annotation quality of the datasets. In addition, to improve system performance and scalability, we will utilize a distributed and parallel platform to fulfill efficient clustering and query generation in order to handle big data in biomedical research.

## Figures and Tables

**Table 1:** Mapping among Clinical Free Text, MedTagger terms and RDF triples.

Clinical Free Text	MedTagger Terms	RDF Triples
1 Patient’s abdominal wound was exacerbated by dressing changes	Abdominal wound exacerbated by dressing change	{abdominal_wound, exacerbated_by, dressing_change}
2 Any problems including increased erythema around the wound	Problems including erythema around wound	{problems, erythema, wound}
3 The residual urine levels drop below certain level	Urine drop below level	{urine, drop, below_level}
4 There is substantial further elevation in patient’s troponins	Place has further elevation in troponins	{place, elevation, troponins}
5 More hypotension requiring initiation of pressor, to achieve satisfactory blood pressure	Hypotension requiring blood	{hypotension, requiring, blood}

**Table 2:** Predicate sharing patterns.

Patterns	Sharing with S and O through P
Subject-Object Share	Si = = Sj & & Oi = = Oj
Subject Share	Si = = Sj
Object Share	Oi = = Oj

**Table 3:** Predicate connectivity patterns.

Patterns	Connecting between S and O through P	

	Symbol	Condition
Path Connectivity	Si → P1 → Oi → P2 → Oj	P1 ≠ P2 && Oi = Sj
Cycle Connectivity	Si → P1 → Oi → P2 → Oj	P1 ≠ P2 && Oi = Sj && Si = Oj

**Table 4:** Definition of colorectal postsurgical complication.

Postsurgical Complication	Description
Abscess/Leak (ABSCESS)	An abscess is a painful collection of pus, usually caused by a bacterial infection. Coloanal anastomoses have the highest rates.
Bleeding (BLEED)	Minor and major bleeding is common in anastomotic complications. Epinephrine and saline retention enemas are used to manage serious bleeding. Surgical intervention is necessary if situation is getting worse.
Deep vein thrombosis (DVT)/pulmonary embolism (PE) (DVTPE)	DVT is a condition wherein a blood clot forms in a vein of the deep system. A piece of the clot can break off and travel through the lung, which can cause heart failure, known as PE.
Ileus (ILEUS)	Ileus is defined as bowel obstruction. For small bowel obstruction, 90–100% sensitivity can be achieved by a CT scan of the abdomen and pelvis.
Myocardial infraction (MI)	Myocardial infarction is commonly known as a heart attack. It occurs during surgery or within 30 days after surgery.
Wound infection (INFECTION)	Wound infections commonly present around the fifth postsurgical day and 5–15% of patients have such complication after colorectal surgery.

**Table 5:** Colorectal surgical cohort.

	# of Subjects	# of Predicates	# of Objects	# of Unique Triples
ABSCESS	63	13	89	220
BLEED	58	13	73	142
DVTPE	19	10	26	32
ILEUS	227	21	204	624
MI	52	12	53	132
INFECTION	26	14	37	60
**Total**	**445**	**83**	**482**	**1210**
